# Mechanical and Corrosion Resistance Enhancement of Closed-Cell Aluminum Foams through Nano-Electrodeposited Composite Coatings

**DOI:** 10.3390/ma12193197

**Published:** 2019-09-29

**Authors:** Yiku Xu, Shuang Ma, Mingyuan Fan, Hongbang Zheng, Yongnan Chen, Xuding Song, Jianmin Hao

**Affiliations:** 1 School of Materials Science and Engineering, Chang’an University, Xi’an 710064, China; 2017131019@chd.edu.cn (S.M.); 2018231004@chd.edu.cn (M.F.); 2017902285@chd.edu.cn (H.Z.); jmhao@chd.edu.cn (J.H.); 2 Key Laboratory of Road Construction Technology and Equipment, MOE, Chang’an University, Xi’an 710064, China; songxd@chd.edu.cn

**Keywords:** aluminum foam, electrodeposition, compression test, corrosion resistance

## Abstract

This work aims to improve the properties of aluminum foams including the mechanical properties and corrosion resistance by electrodepositing a SiC/TiN nanoparticles reinforced Ni–Mo coating on the substrate. The coatings were electrodeposited at different voltages, and the morphologies of the coating were detected by SEM (scanning electron microscope) to determine the most suitable voltage. We used XRD (x-ray diffraction) and TEM (transmission electron microscope) to analyze the structure of the coatings. The aluminum foams and the substrates on which the coatings were electrodeposited at a voltage of 6.0 V for different electrodeposition times were compressed on an MTS (an Electro-mechanical Universal Testing Machine) to detect the mechanical properties. The corrosion resistance before and after the electrodeposition experiment was also examined. The results showed that the coating effectively improved the mechanical properties. When the electrodeposition time was changed from 10 min to 40 min, the W_v_ of the aluminum foams increased from 0.852 J to 2.520 J and the **σ**_s_ increased from 1.06 MPa to 2.99 MPa. The corrosion resistance of the aluminum foams was significantly improved after being coated with the Ni–Mo–SiC–TiN nanocomposite coating. The self-corrosion potential, pitting potential, and potential for primary passivation were positively shifted by 294 mV, 99 mV, and 301 mV, respectively. The effect of nanoparticles on the corrosion resistance of the coatings is significant.

## 1. Introduction

Aluminum foams have the characteristics of both metal materials and foam materials due to their special structure. They are functional materials with the properties of both structural materials and functional materials [1,2,3,4]. Aluminum foams have a unique stress–strain curve including a linear elastic region, a plastic collapse region, and a densification region, which makes aluminum foam materials suitable for use as an energy absorber [5]. Due to its excellent properties including light weight, high sound absorption and insulation performance, heat resistance, and high cushioning performance, it is widely used in sound absorption and sound insulation structures such as sound barriers and sound insulation boards, and for energy absorption and collision protection in automobiles [6,7,8,9]. However, the high porosity of aluminum foam significantly lowers its mechanical strength. When applied in an engineering field, it often fails prematurely, which greatly limits its potential range of applications. For example, when an aluminum foam is used for sound insulation and heat transfer in an environment that requires a certain load, the aluminum foam will fail and be crushed after the load acts on it for a long period of time [10]. The seawater, micro-organisms, and salt spray in a marine environment can corrode aluminum foams when they are used in marine transportation applications. In order to expand the range of uses of aluminum foams and make them better for practical applications, surface modification methods are used to simultaneously improve their mechanical properties and corrosion resistance. An aluminum foam with a high energy absorption capacity should have a longer and higher stress platform. At present, using the same material to thicken the foam pillar is a common method for improving the energy absorption capacity [11,12,13,14]. However, this method has certain limitations. When the pillar of the aluminum foam is thickened, the platform stress will be improved, but the aluminum foam will be dense [15,16,17]. Densification may limit the ability of aluminum foams to absorb energy. At present, the commonly used surface modification methods for aluminum foams include micro-arc oxidation, anodization, electro-less plating, sol–gel deposition, and electrodeposition [18,19,20]. Of these methods, electrodeposition is widely used because it is simple, low cost, and easy to control.

At present, there are a number of reports on the use of deposited layers to enhance the properties of aluminum foams. Yuttanant Boonyongmaneerat et al. [21] electrodeposited a nanocrystalline Ni–W coating on open-cell aluminum foams to improve their properties including compressive strength and energy abortion. Zhendong Li et al. [22] confirmed that a thermally evaporating Zn film could significantly enhance open-cell aluminum foams and increase their yield strength. Liu Huan et al. [10] studied the enhancements that a Ni coating could provide to closed-cell aluminum foams. It demonstrated that a Ni coating could improve the properties of aluminum foams including both the mechanical and corrosion resistance properties. Jiaan Liu et al. [23] showed that the corrosion resistance of closed-cell aluminum foams could be improved by an electro-less Ni–P coating. Due to the excellent mechanical properties, corrosion resistance, and wear resistance of the Ni–Mo coating, it often used as an protective coating [24,25,26]. SiC nanoparticles have a high degree of hardness, wear resistance, and thermal stability, and TiN nanoparticles have a high degree of hardness, high strength, and corrosion resistance [27,28,29]. As far as we know, no research has been done on the use of a Ni–Mo coating and a duplex nanoparticles reinforced Ni–Mo coating to enhance the properties of closed-cell aluminum foams.

In this work, the influence of a Ni–Mo coating and a duplex nanoparticles reinforced Ni–Mo coating on the mechanical properties and corrosion resistance of closed-cell aluminum foams was studied. The effects of electrodeposition voltage and electrodeposition time on the morphology, mechanical properties, and corrosion resistance of the closed-cell aluminum foams were investigated. The deposition mechanism of the duplex nanoparticles reinforced Ni–Mo coating is also discussed.

## 2. Materials and Methods

### 2.1. Samples and Solution

We used the method of melt foaming to prepare the closed-cell aluminum foams in this experiment. The density of the samples was 0.2 g/cm^3^. The pore diameter was 4 mm. We used an electrical discharge machine to reduce the sample’s dimensions to 20 mm × 20 mm × 9 mm.

To create a good bond between the substrate and the coating, the aluminum foam was pretreated before the electrodeposition experiment. The aluminum foam sample was immersed in a 10~15% H_2_SO_4_ solution at 60 °C for 1–3 min. After immersion, the oil was removed. Then, a 5% NaOH solution was used to remove the Al_2_O_3_ film from the surface of the samples. The sample was immersed for 2 min. Finally, the aluminum foam was immersed for 5 min in a 10% HNO_3_ solution. The corrosion products were removed and activated. After each of the steps was completed, the sample was washed with distilled water to prevent the pretreatment liquid from being contaminated. After the pretreatment steps were completed, the aluminum foam sample was placed in the electrolyte immediately to prevent it from being oxidized in the air.

Table 1 shows the electrolyte components that were used in this experiment. The electrolyte was composed of analytically pure reagent and distilled water. The added SiC and TiN nanoparticles (Shanghai Chaowei Nanotechnology Co. Ltd., Nanxiang Hi-Tech Industrial Park, Jiading District, Shanghai) both had a mean particle diameter of 20 nm and purity of 99 wt.%. Since nanoparticles tend to agglomerate in the electrolyte, SDS was chosen as the dispersing agent. The electrolyte was subjected to ultrasonic treatment for 2 h and the electrolyte was stirred using a magnetic stirrer with a speed of 300 rpm during the electrodeposition experiment. Electrolyte (200 mL) was placed in a bath, pure nickel plate (99.99 wt.%) was used as the anode, and the aluminum foam sample was used as the cathode. The anode and the cathode had a distance of 30 mm between them. 

### 2.2. Morphology Investigation

The surface morphology and a cross-section of the coating were observed using a Hitachi S4800 field scanning electron microscope (SEM, Hitachi, Ltd., Tokyo, Japan). The elements were analyzed by energy dispersive x-ray spectroscopy. The structure of the coating was examined by D8 ADVANCE x-ray diffraction (XRD, Bruker, Karlsruhe, Germany). Cu-k_α_ radiation was selected, and the 2θ range was 20~80°. In order to further analyze the specific structure of the nanocomposite coating and the distribution of nanoparticles, the coating was examined by an FEI Talos F200X transmission electron microscope (TEM, FEI™, Hillsboro, OR, USA) including high-resolution TEM (HR-TEM) and selected area electron diffraction (SAED).

### 2.3. Properties Investigation

An electrodeposited aluminum foam with the dimensions of 20 mm × 30 mm × 40 mm was subjected to a quasi-static compression test on an MTS (an Electro-mechanical Universal Testing Machine, American MTS Corporation, MN, USA) with a selected load of 10 KN, a compression speed of 5 mm/min, and a compression amount greater than 70%.

The corrosion resistance of the sample at room temperature was measured by the three-electrode working system. In this experiment, a 3.5 wt% NaCl solution was used as the etching solution. The working electrode was the aluminum foam sample, the reference electrode was the saturated calomel electrode, and the counter electrode was the platinum plate electrode. The selected voltage range was −2 to 1 V and the scan rate was 2 mV/s.

The samples were placed in an immersion test for 120 h to measure the corrosion rate at 25 °C. The immersion solution was a 3.5 wt% NaCl. The samples were weighed to calculate the mass loss every 24 h. Distilled water was used to rinse the samples, and they were dried thoroughly before each weighing. The weight of a sample was expressed as the average of three measurements. The analytical balance that was used to weigh the samples had an accuracy of 0.01 mg. 

## 3. Theoretical Models

Electrodeposition of metals and alloys refers to the reduction of metal ions from an electrolyte, where electrons (*e*) are provided by an external power supply. The reaction time and the current can optimize the thickness of a coating. Molybdenum cannot be electrodeposited from the electrolyte solution, but the co-deposition of nickel and molybdenum can be achieved using sodium citrate as an inducer. During the electrodeposition of Ni–Mo composite coatings on an aluminum foam, the following chemical reactions occur at the cathode and anode [30]:

Anode:

(1) $$\mathrm{Ni}-2e\to {\mathrm{Ni}}^{2+}$$

Cathode:

(2) $${\mathrm{Ni}}^{2+}+2\;e\to \mathrm{Ni}$$

(3) $${{\mathrm{MoO}}_{4}}^{2-}+2H_{2}O+2e\to {\mathrm{MoO}}_{2}+4{\mathrm{OH}}^{-}$$

(4) $${\mathrm{NiCit}}^{-}+{\mathrm{MoO}}_{2}\to {{\left[{\mathrm{NiCitMoO}}_{2}\right]}^{-}}_{ads}$$

(5) $${{\left[{\mathrm{NiCitMoO}}_{2}\right]}^{-}}_{ads}+2H_{2}O+4e\to \mathrm{Mo}+{\mathrm{NiCit}}^{-}+4{\mathrm{OH}}^{-}$$

**Figure 1:**
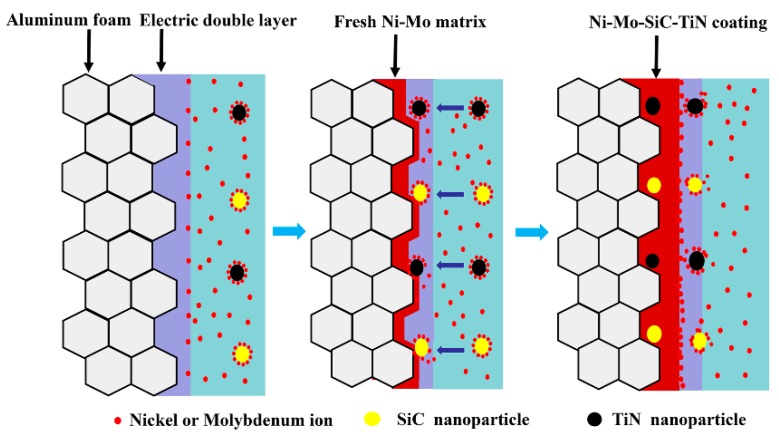
A schematic diagram representing the electrodeposition process of a duplex nanoparticles reinforced Ni–Mo coating.

With respect to the co-deposition of nanoparticles with a Ni–Mo matrix, the processes include three main steps, as illustrated in Figure 1. According to Gugliemi’s absorption model, Ni ions and Mo ions in the electrolyte solution are first adsorbed on the nanoparticles to form Ni/Mo ionic clouds. Under the electric field force, metal ions and ionic clouds move toward the cathode and are tightly adsorbed on the aluminum foams. Then, the Ni and Mo ions adsorbed on the surface of the nanoparticles are reduced partially at the surface of the foam. Simultaneously, nanoparticles are trapped by the metal matrix and embedded in the Ni–Mo plating layer.

Based on a theoretical model of Cu electrodeposition, the model of the electrodeposited Ni–Mo alloy coating in this experiment is now described [31].

The plating deposition rate is expressed by P%, and its expression is:

(6) $$P\%=[{\left(P_{2}\right)}_{i}-P_{1}{}_{i}]/P_{1}{}_{i}$$

where P_1_ indicates the mass of the substrate before the electrodeposition experiment; P_2_ indicates the mass of the aluminum foam covered with a coating; and *i* indicates the sample number. P% is the ratio of the mass of the aluminum foam covered with a Ni–Mo coating to the mass of the aluminum foam before electrodeposition.

(7) $$P\%=\frac{M_{\mathrm{NiMo}}}{\rho *V_{i}}=({\mathrm{MM}}_{\mathrm{NiMo}}{*n}_{\mathrm{NiMo}})/\left(\rho {*V}_{i}\right)$$

where M_NiMo_ is the mass of the deposited Ni–Mo alloy coating; MM_Ni–Mo_ is the molar mass of Ni–Mo alloy; and *ρ* and *V_i_* indicate the density and volume of the aluminum foam before the electrodeposition experiment, respectively.

The Ni–Mo alloy that was formed in this experiment is a Ni–Mo solid solution. When 1 mole of Ni–Mo alloy coating is deposited, 14 moles of electron are required. Then, P% also can be expressed as:

(8) $$P\%=\left({\mathrm{MM}}_{\mathrm{NiMo}}*n_{e}\right)/\;\left(14*\rho *V_{i}\right)$$

where *e* is the electric charge of an electron.

It is known that *n_e_* = q/(N_a_ ∗ e), q = *i* ∗ *t*. Then,

(9) $$P\%={\mathrm{MM}}_{\mathrm{NiMo}}/(14{*N}_{a}*e]*[\left(i*t\right)/\left(\rho *V_{i}\right)$$

where *t* is the electrodeposition time (in minutes); *N_a_* is Avogadro’s number with a value of 6.02 × 10^23^; MM_NiMo_ is 331 g/mol; and *e* is 1.6 × 10^−19^ C. Then,

(10) $$P\%=2.45\times 10^{-4}*\left[\left(i*t\right)/\left(\rho *V_{i}\right)\right].$$

As this experiment was carried out under a certain voltage, the expression is written as

(11) $$P\%=2.45\times 10^{-4}*\left[\left(u*t\right)/\left(\rho *V_{i}*r\right)\right]$$

where *u* is the electrodeposition voltage and *r* is the total resistance.

The relationship between the deposition rate of a Ni–Mo coating, the electrodeposition voltage *u*, and the time *t* can be obtained by Equation (11), and the P% that is obtained by experiments can be verified using theoretical calculations.

## 4. Results and Discussion

### 4.1. Coating Characterization

**Figure 2:**
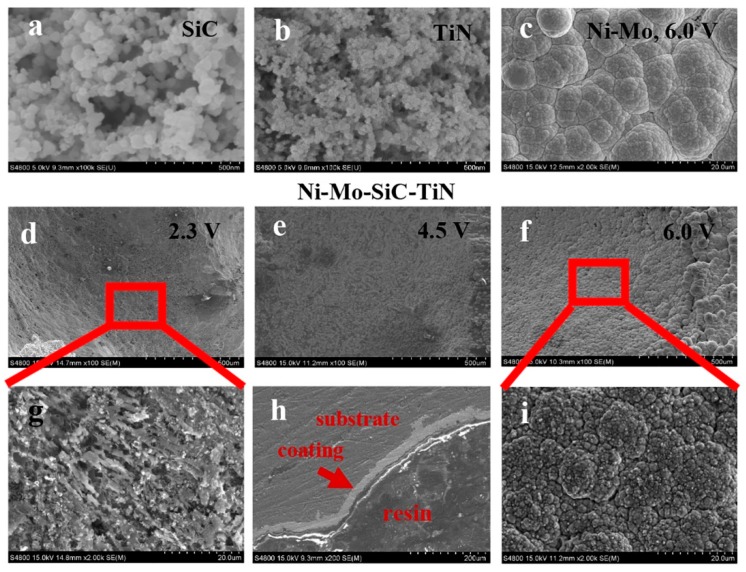
SEM (scanning electron microscope) images of (**a**) SiC nanoparticles, (**b**) TiN nanoparticles, (**c**) the Ni–Mo coating, and the duplex nanoparticles reinforced Ni–Mo coating electrodeposited (**d**) at 2.3 V, (**e**) 4.5 V, and (**f**) 6.0 V. The enlarged SEM images of the duplex nanoparticles reinforced Ni–Mo coating (**g**) at 2.3 V, (**i**) 6.0 V; and (**h**) morphology of a cross-section of the duplex nanoparticles reinforced Ni–Mo coating.

Figure 2b show the SEM images of the two kinds of nanoparticles with an original size of approximately 20 nm. As can be seen, both kinds of nanoparticles were agglomerated due to the surface effect.

Figure 2d–f show the morphologies of the electrodeposited duplex nanoparticles reinforced Ni–Mo coatings, applying electrodeposition voltages ranging from 2.5 V to 6.0 V, respectively. It has been reported that nanoparticles can make a coating have a finer grain and a higher microhardness. In accordance with this, SiC- and TiN-reinforced coatings have structures with finer grain sizes than Ni–Mo composite coatings. As the voltage increased, coating particles were gradually formed and completely covered the substrate. When the voltage was increased to 6.0 V, a uniform coating was prepared on the aluminum foam. The SEM image shown in Figure 2i revealed that the surface of the coating had nanoparticles dispersed upon it. At the voltage of 6.0 V, a nodular and homogenous Ni–Mo coating was also obtained. It is known that a larger electrodeposition voltage can increase the nucleation driving force, so plating particles are formed. The metal ion deposition rate was sufficiently high to form a uniform and dense coating on the substrate at the voltage of 6.0 V. Figure 2h shows the morphology of a cross-section of aluminum foam that was subject to electrodeposition for 10 min at 6.0 V. The coating had a thickness of about 25 μm. The bond between the plating layer and the substrate was good, the thickness of the plating layer was relatively uniform, and there were no cracks or discontinuities.

**Figure 3:**
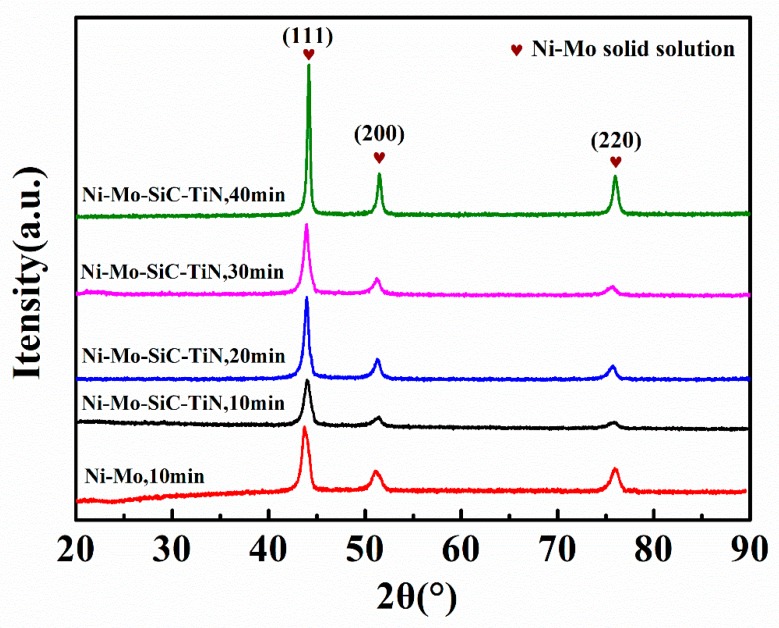
XRD (x-ray diffraction) patterns of coatings electrodeposited by different times.

Figure 3 shows the XRD patterns of the coatings. The body-centered cubic structure that corresponds to nickel’s (111), (200), and (220) diffraction peaks. No diffraction peak of molybdenum was detected, indicating that the nickel atom and the molybdenum atom existed in the form of a Ni–Mo solid solution. The nanoparticles did not change the structure of the Ni–Mo coating. In the XRD patterns, there were no diffraction peaks related to nanoparticles. This is mainly because the size of the nanoparticles was too small, their content too low, and the distribution was uniform [32]. The intensity of the peaks of the XRD patterns of the coatings electrodeposited at 6.0 V for different times were different. We used the Scherrer formula to calculate the crystallite size:

(12) D=Kλ/(βCOSθ)

where λ represents the wavelength of the x-ray (0.15406 nm); K is the Scherrer constant (0.9); β is the full width of the reflection line at half maxima; and θ is a Bragg diffraction angle.

Table 2 shows the results. As the electrodeposition time increased, the grains of the coatings accumulated and the crystallite size increased. Comparing the crystallite size of the Ni–Mo coating to that of the duplex nanoparticles reinforced Ni–Mo coating, it can be seen that the nanoparticles decreased the crystallite size of the coating. Nanoparticles can inhibit the grain growth because they provide nucleation dots.

**Figure 4:**
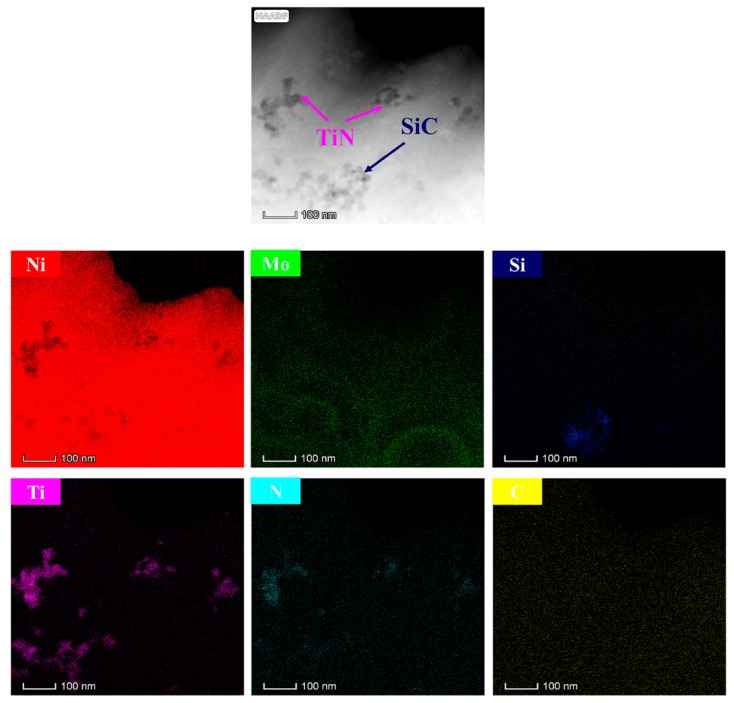
EDS (energy dispersive spectrometer) element mapping of the Ni–Mo–SiC–TiN nanocomposite coating.

Figure 4 shows the EDS (energy dispersive spectrometer) elements mapping of the duplex nanoparticles reinforced Ni–Mo coating. Ni, Mo, Si, C, Ti, and N elements were detected. The existence of Si, C, Ti, and N elements indicates that duplex nanoparticles were successfully electrodeposited in the Ni–Mo composite coating. The EDS element mapping demonstrates the specific distribution of duplex nanoparticles. Nanoparticles were uniformly dispersed in the coating, but partial agglomeration occurred. Since the coating used for EDS (energy dispersive spectrometer) detection was a 100 nm thin layer, the distribution of nanoparticles inside the coating can be known.

**Figure 5:**
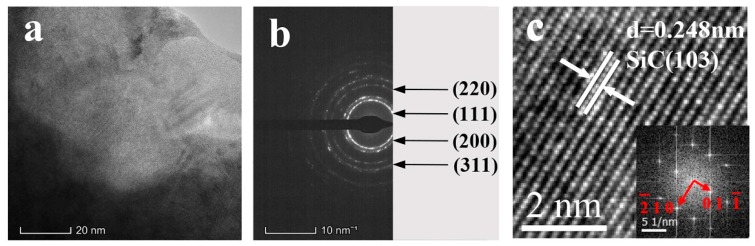
(**a**) Bright field image of the duplex nanoparticles reinforced Ni–Mo coating; (**b**) diffraction ring of the metal matrix; and (**c**) HR-TEM image of the nanoparticles.

Figure 5a presents the TEM images under a bright field. From the images, it was found that the nanoparticles were tightly embedded in the Ni–Mo metal matrix and there were no voids between them. The interface between the nanoparticles and the Ni–Mo metal matrix was clear and there were no harmful interfacial reaction products. The selected area electron diffraction rings in Figure 5b correspond to the (111), (200), (220), and (311) crystal faces of the nickel–molybdenum solid solution, respectively. The fast Fourier transform (FFT) and inverse FFT of the nanoparticles in Figure 5a are shown in Figure 5c. The nanoparticles were proven to be 6H–SiC, which have a hexagonal closed-packed (HCP) structure with a Lattice constant of 3.08 Å.

### 4.2. Mechanical Behavior

**Figure 6:**
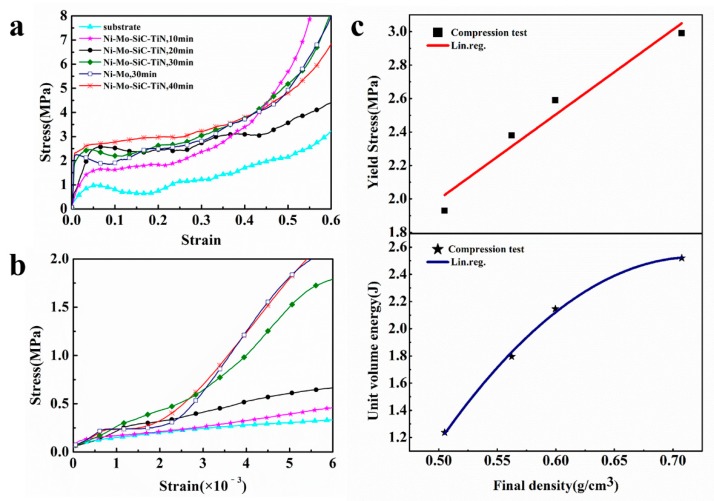
(**a**) Stress–strain curves of aluminum foam and aluminum foam subject to electrodeposition at the voltage of 6.0 V; (**b**) the enlarged elastic region of (**a**). (**c**) the yield strength and unit volume energy in function of the final density of samples.

Figure 6a shows the stress–strain curves of the aluminum foam and the aluminum foams subjected to electrodeposition for different times. The enlarged elastic region of the curve is shown in Figure 6b. The stress–strain curve of the aluminum foam includes three parts: an elastic deformation stage, a yield stage, and a densification stage. In the initial stage of the compression experiment, the stress increased as the strain increased. The relationship between stress and strain was linear. When the curve entered the yield stage, the stress appeared to be small or substantially constant as the strain increased. In the densification stage, since the pores inside the aluminum foam burst and collapsed, the stress increased sharply at this stage and the strain remained substantially unchanged.

Compared with the aluminum foam matrix, the elastic modulus and the platform stress of the aluminum foams after the coating was deposited were improved. When the strain remained the same, the stress of the aluminum foam after electrodeposition was larger than that of the aluminum foam substrate. There are two main reasons for the increase in strength and elastic modulus of aluminum foams with electrodeposited coatings. The first reason is that, due to the particularity of the structure of the aluminum foam, the deformation of the aluminum alloy during the compression experiment was not synchronized, resulting in separation of the coating from the substrate. The second reason is the friction and extrusion between the coating and the substrate. The stress–strain curve also showed that the coating increased the energy absorption of the aluminum foam.

The density of an aluminum foam affects its mechanical strength. An increase in density will increase the compressive properties. The density is related to the electrodeposition time. The electrodeposition time determines the quality of the coating that is deposited on the substrate, so the quality affects the mechanical strength of the substrate. Table 3 lists the coefficient of variation P% of different electrodeposition times. The resistance, which includes the external contact resistance *r*_1_, the solution resistance *r*_2_, and the resistance of the cathode film r_3_ during the electrodeposition process, are all uncertain. The resistance at 10 min of electrodeposition was used as the resistance in this experiment.

The coefficient of variation of 20 min, 30 min, and 40 min of electrodeposition, as calculated by the established electrodeposition theoretical model, was 21.35%, 33.76%, and 42.16%, respectively, while the P% obtained from the experiment was 23.5%, 38.8%, and 53.4%, respectively. Density of Ni–Mo–SiC–TiN coatings does not obviously change with deposition time. As the deposition time increased, the error between the theoretical model and the results obtained from deposition rate increased. The main reason for this is that an increase in the electrodeposition time will cause a large change in resistance.

Table 3 also lists the density, yield strength, densification strain, and W_v_ of the aluminum foam after the electrodeposition experiments. W_v_ represents the energy absorbed per unit volume when the aluminum foam is deformed. When comparing the compression properties of the samples after different electrodeposition times, it was found that the stress–strain curves of aluminum foams moved upward with the increase of electrodeposition time. This is mainly because an increase in electrodeposition time will increase the quality of the coating on the aluminum foam. The quality of the coating on the surface increases the strength and stiffness of the aluminum foam. From the stress–strain curve, it can be seen that the curve appeared to fluctuate in the stress platform stage, which is due to the instability of the aluminum foam. This can be attributed to the non-uniformity of the aluminum foam’s cell structure and its rough surface. When the stress–strain curve passes the linear elastic phase, the stress tends to decrease; the reasons for this are discussed in the literature [33,34].

The stress remained almost constant as the strain increased in the stress platform stage, which allowed the sample to absorb a large amount of energy during the compression process. Figure 6 shows the absorbed energy per unit volume of the aluminum foam during the quasi-static compression experiment in the stress–strain curve. Its calculation expression is [35]

(13) $$W_{v}=\int_{0}^{\varepsilon_{D}}\sigma \left(\varepsilon \right)d\varepsilon$$

where ε_D_ represents the densification strain, which corresponds to a sharp rise in stress during compression because the aluminum foam is crushed and deformed and the cell structure completely collapses, and **σ**(ε) represents the stress.

Gibson et al. proposed the following relationship between the densification strain of closed-cell aluminum foam, $\varepsilon_{D}$ [36], and the relative density $\bar{\rho }$:

(14) $$\varepsilon_{D}=1-1.4\;\bar{\rho }$$

where $\bar{\rho }$ is the ratio of the apparent density *ρ* of the aluminum foam to the density *ρ*_s_ (2.70 g/cm^3^) of the aluminum foam matrix.

The relationship between the W_v_ and the apparent density *ρ* of aluminum foams is obtained from the above two formulas:

(15) $$W_{v}=\int_{0}^{1-0.518\rho }\sigma \left(\varepsilon \right)d\varepsilon .$$

The relationship indicates that W_v_ is related to the density of aluminum foams.

The specific relationship between the density and mechanical properties of samples was explored after the electrodeposition experiments, the density of aluminum foams after the electrodeposition of a coating between the W_v_, and yield strength **σ_s_**were respectively fitted. The fitting results are shown in Figure 6c, d. The relationship between the unit volume energy absorption W_v_ and the density *ρ* is

(16) $$W_{v}=a+b_{1}\rho +b_{2}\rho^{2}.$$

From the fitting results, the value of a, b_1_, and b_2_ is −12.05942 ± 1.72093, 40.51736 ± 5.70059, and −28.13555 ± 4.65988, respectively. The value of the correlation coefficient R^2^ is 0.99376.

The relationship between the yield strength **σ**_s_ and the density *ρ* is

(17) $$\sigma_{s}=d+c\rho .$$

From the fitting results, the value of d, c, and the correlation coefficient R^2^ is −0.5350 ± 0.44692, 5.0664 ± 0.74711, and 0.93748, respectively.

Due to the particular structure of each cell of the aluminum foams and the differences in the deposition rate, the data shown in Figure 6c are relatively discrete.

When the aluminum foams were subjected to electrodeposition for 10 min, 20 min, 30 min, and 40 min, W_v_ was quadratic with *ρ*, and **σ**_s_ increased linearly with *ρ*. Comparing the mechanical properties of the substrates, which were coated with a Ni–Mo coating and a duplex nanoparticles reinforced Ni–Mo coating, the addition of nanoparticles only slightly increased W_v_ and **σ**_s_. This limited enhancement of the compressive properties is due to the small amount of nanoparticles in the Ni–Mo coatings.

### 4.3. Corrosion Resistance

**Figure 7:**
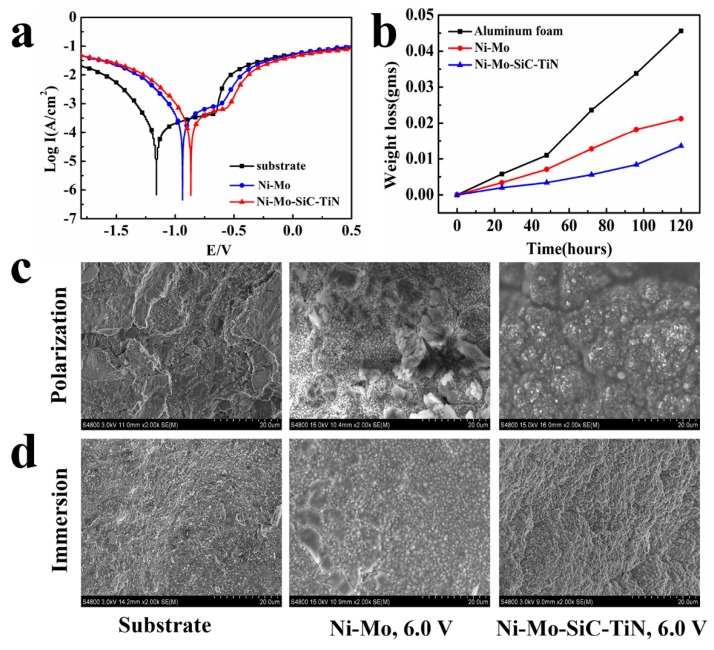
The polarization curves of the samples (**a**) and weight loss versus time curves after the immersion test (**b**); SEM images of the substrate and coatings after the polarization test (**c**) and immersion test (**d**).

The corrosion resistance of the aluminum foam and the aluminum foams with an electrodeposited Ni–Mo coating and a duplex nanoparticles reinforced Ni–Mo coating was detected. The obtained polarization curves are shown in Figure 7a. Table 4 lists the corrosion parameters extracted from the polarization curves. After the Ni–Mo coating was electrodeposited on the aluminum foam, the corrosion potential of the aluminum foam was positively shifted from −1160 mV to −937 mV and the corrosion current density decreased from 4.48 × 10^−5^ A/cm^2^ to 3.90 × 10^−5^ A/cm^2^. The pitting potential and the potential for primary passivation were positively shifted by 48 mV and 225 mV, respectively. The positive shift of the Zero current potential was due to changes in the hydrogen evolution reduction process. Both the aluminum foam and the aluminum foam with an electrodeposited Ni–Mo coating formed passive films. The aluminum foam formed a passive film because of the oxide layer. The oxygen-rich surface reacted with the etching solution to form an adsorption layer. The adsorption layer prevented contact of the etching solution with the surface of the plating layer to prevent the hydration of nickel, which is the first step in the formation of a passive nickel film on the surface of the aluminum foam covered with the Ni–Mo coating.

Compared with the substrate, the corrosion resistance of the samples after electrodeposition was greatly improved. The Ni–Mo coating was found to effectively protect the aluminum foam from corrosion. In order for a corrosive liquid to have a corrosive effect on the aluminum foam’s substrate, the passivation film on the surface of the aluminum foam must first be destroyed. The $Cl^{-}$ in the etching solution was found to easily pass through the passivation film due to the small radius and adsorb on the samples to hinder the adsorption of oxygen. The cations in the passivation film combined with the $Cl^{-}$ to form a soluble chloride. The substrate was partially exposed due to the local corrosion. The aluminum foam had a galvanic effect with the oxide film to form a corrosive micro-battery. The matrix and the impurity elements Ca, Ti, and Si, which were contained in the aluminum foam, also formed a corrosive micro-battery. This resulted in an uneven accumulation and distribution of $Cl^{-}$, which exacerbated the local corrosion.

The coating was able to effectively protect the aluminum foam matrix mainly because the electrodeposited coating could separate the aluminum foam matrix from the etching solution so the Ni-Mo coating had an initial corrosion. The amorphous Ni–Mo alloy coating had good corrosion resistance, and the Mo element could easily form an inert oxide with oxygen in solution to prevent further corrosion of the coating [37,38]. The plating layer was uniform and compact. The coating had a thickness of about 25 μm. The pinholes and cracks in the plating layer were reduced. These all made the distribution of $Cl^{-}$ become uniform. The corrosion on the surface of the coating was relatively uniform. Then, there was a better corrosion resistance.

From the polarization curves of the samples, corrosion parameters can be obtained. The corrosion potential was shifted from –0.937 V for the Ni–Mo coating to –0.866 V for the duplex nanoparticles reinforced Ni–Mo coating, and the corrosion current density was reduced from 3.90 × 10^−5^ A/cm^2^ to 2.72 × 10^−5^ A/cm^2^. This change illustrates that the SiC and TiN nanoparticles can have an improvement on the corrosion resistance of the Ni–Mo coating. The reason for this is that these two kinds of nanoparticles are inert nanoparticles that have a certain degree of corrosion resistance. Dispersed nanoparticles can enhance the corrosion resistance of the coating because the nanoparticles can block the etching solution and the coating from coming into contact.

Figure 7c shows the SEM of the sample after the polarization experiment in corrosive solution. The aluminum foam was severely corroded, and there were many corrosion products and corrosion pits on the aluminum foam. An EDS spectrum analysis was performed on the corrosion surface, and the oxygen content in the corrosion product was found to be 17.04 wt.%. Compared with the aluminum foam, the Ni–Mo coating provided better protection to the substrate. Corrosion occurred on the surface of the coating when corrosion occurred. Local corrosion cracks could be observed, and the oxygen content in the corrosion products decreased to 8.03 wt.%. After adding duplex nanoparticles, the number of corrosion products was significantly reduced. This is because the two inert types of nanoparticles protected the matrix coating. Nanoparticles filled the voids in the matrix coating and improved the compactness of the coating. The smaller contact area effectively reduced the corrosion rate. The low content (4.69 wt.%) of oxygen also indicated an improvement in corrosion resistance.

Figure 7d shows the corrosion morphologies of the aluminum foam, the Ni–Mo coating, and the duplex nanoparticles reinforced Ni–Mo coating deposited at the voltage of 6.0 V after the immersion test. There are many corrosion pits on the surface of the substrate. The aluminum foam was obviously corroded because there was no protection of the coatings. The Ni–Mo coating was slightly corroded, and only a few corrosion products existed. Almost no corrosion was observed on the surface of the duplex nanoparticles reinforced Ni–Mo coating due to the protection of the nanoparticles.

Table 4 lists the corrosion rates of different samples. Compared with the aluminum alloy matrix, the corrosion rates of the Ni–Mo coating and the duplex nanoparticles reinforced Ni–Mo coating were improved by 51.9% and 72.5%, respectively.

## 5. Conclusions

1. A uniform and dense duplex nanoparticles-reinforced Ni–Mo coating with a thickness of 25 μm was obtained by electroplating on the aluminum foam surface for 10 min at 6.0 V. The bond between the substrate and the coating was good.
2. The duplex nanoparticles reinforced Ni–Mo coating had a structure of FCC. The crystallite size of the Ni–Mo coatings was decreased from 13.31 nm to 12.14 nm after adding the duplex nanoparticles. The results indicate that increasing the electrodeposition time can effectively enlarge the crystallite size.
3. After the aluminum foams were coated with a duplex nanoparticles-reinforced Ni–Mo coating, there was a significant improve in the mechanical properties of the aluminum foams. When the electrodeposition time was 40 min, the W_v_ of the aluminum foam increased from 0.852 J to 2.520 J, and the **σ**_s_ increased from 1.06 MPa to 2.99 MPa. The addition of nanoparticles made a limited improvement to the mechanical properties.
4. The duplex nanoparticles-reinforced Ni–Mo coating was found to have better corrosion resistance. Compared to the aluminum foams, the self-corrosion potential, the pitting potential, and the potential for primary passivation were positively shifted by 294 mV, 99 mV, and 301 mV, respectively. The corrosion rate of the aluminum foam covered with a Ni–Mo coating was reduced by 51.9%. After adding nanoparticles, the corrosion rate was reduced by 72.5%. The nanoparticles obviously improved the corrosion resistance.

## Figures and Tables

**Table 1:** Components of the electrolyte for the electrodeposition of the Ni–Mo–SiC–TiN coating.

Bath Composition	Concentration	Purpose
NiSO_4_·6H_2_O	0.27 mol·${\mathrm{dm}}^{3}$	Ni source
Na_2_MoO_4_·2H_2_O	0.032 mol·${\mathrm{dm}}^{-3}$	Mo source
Na_3_C_6_H_5_O_7_·2H_2_O	0.52 mol·${\mathrm{dm}}^{-3}$	Complexing agent
NH_4_Cl	0.65 mol·${\mathrm{dm}}^{-3}$	Buffer
SDS	0.1 g·${\mathrm{dm}}^{-3}$	Surfactant
SiC	5 g·${\mathrm{dm}}^{-3}$	Composite phase
TiN	5 g·${\mathrm{dm}}^{-3}$	Composite phase

**Table 2:** Crystallite size of different coatings electrodeposited at 6.0 V.

Coatings (Electrodeposition Time)	Crystallite Size (nm)
Ni–Mo (10 min)	13.31
Ni–Mo–SiC–TiN (10 min)	12.14
Ni–Mo–SiC–TiN (20 min)	17.01
Ni–Mo–SiC–TiN (30 min)	20.36
Ni–Mo–SiC–TiN (40 min)	24.96

**Table 3:** Characteristics of different samples.

Samples	Deposition Time t (min)	$\mathrm{Initial}\;\mathrm{Density}\;\rho_{0}\;(g/{\mathrm{cm}}^{3})$	$\mathrm{Final}\;\mathrm{Density}\;\rho \;(g/{\mathrm{cm}}^{3})$	Coefficient of Variation P% (%)	Yield Strength σ_s_ (MPa)	Elastic Modulus (MPa)	W_v_ (J)
Substrate	0	/	/	/	1.06	43.67	0.852
Ni–Mo–SiC–TiN	10	0.4562	0.5051	10.7	1.93	54.39	1.237
Ni–Mo–SiC–TiN	20	0.4553	0.5623	23.5	2.38	106.27	1.795
Ni–Mo–SiC–TiN	30	0.4320	0.5996	38.8	2.59	248.63	2.146
Ni–Mo	30	0.4326	0.5976	38.2	2.52	240.46	2.069
Ni–Mo–SiC–TiN	40	0.4612	0.7075	53.4	2.99	344.75	2.520

**Table 4:** The corrosion parameters extracted from the polarization curves and weight loss vs. time curves.

Passivation Parameters	Substrate	Ni–Mo	Ni–Mo–SiC–TiN
Potential for primary passivation (E_pp_, mV)	−1130	−905	−829
Breakdown potential (E_b_, mV)	−653	−605	−554
Corrosion potential (E_corr_, mV)	−1160	−937	−866
Corrosion current density (I_corr_, A/cm^2^)	4.48 × 10^−^^5^	3.90 × 10^−^^5^	2.72 × 10^−^^5^
$\beta_{a}$ (mV/decade)	69.08	47.14	33.32
$\beta_{c}$ (mV/decade)	25.47	29.88	40.45
Corrosion rate (g/cm^2^·h)	3.8643 × 10^−^^4^	1.8583 × 10^−^^4^	1.0643 × 10^−^^4^

## References

[1] Banhart J., Baumeister J., Weber M. (1996). Damping properties of aluminum foams. Mater. Sci. Eng. A.

[2] Uzun A., Karakoc H., Gokmen U., Cinici H. (2017). Investigation of mechanical properties of tubular aluminum foams. Int. J. Mater. Res..

[3] Gong L., Kyriakides S., Jang W.Y. (2005). Compressive response of open-cell foams. Part I: Morphology and elastic properties. Int. J. Solids Struct..

[4] Song H.W., Fan Z.J., Yu G., Wang Q.C., Tobota A. (2005). Partition energy absorption of axially crushed aluminum foam-filled hat sections. Int. J. Solids Struct..

[5] Wang W., Burgueño R., Hong J.W., Lee I. (2013). Nano-deposition on 3-d open-cell aluminum foam materials for improved energy absorption capacity. Mater. Sci. Eng..

[6] Kim A., Hasan M.A., Nahm S.H. (2005). Evaluation of compressive mechanical properties of Al-foams using electrical conductivity. Compos. Struct..

[7] Rajendran R., Sai K.P., Chandrasekar B., Gokhale A., Basu S. (2008). Preliminary investigation of aluminium foam as an energy absorber for nuclear transportation cask. Mater. Des..

[8] Liang L.S., Yao G.C., Wang L., Ma J., Hua Z.S. (2010). Sound absorption of perforated closed-cell aluminum foam. Chin. J. Nonferrous Met..

[9] Zhang C.J., Feng Y., Zhang X.B. (2010). Mechanical properties and energy absorption properties of aluminum foamfilled square tubes. Trans. Nonferrous Met. Soc. China.

[10] Liu H., Yao G.C., Cao Z.K., Hua Z.K., Shi J.C. (2012). Properties of aluminum foams with electrodeposited Ni coatings. Chin. J. Nonferrous Met..

[11] Marchi C.S., Mortensen A. (2001). Deformation of open-cell aluminum foam. Acta Mater..

[12] Jung A., Natter H., Diebels S., Lach E., Hempelmann R. (2011). Nanonickel Coated Aluminum Foam for Enhanced Impact Energy Absorption. Adv. Eng. Mater..

[13] Jung A., Lach E., Diebels S. (2014). New hybrid foam materials for impact protection. Int. J. Impact Eng..

[14] Yi F., Zhu Z., Zu F., Hu S., Yi P. (2001). Strain rate effects on the compressive property and the energy-absorbing capacity of aluminum alloy foams. Mater. Charact..

[15] Evans A.G., Hutchinson J.W., Ashby M.F. (1998). Multifunctionality of cellular metal systems. Prog. Mater. Sci..

[16] Gibson L. (2000). Mechanical behavior of metallic foams. Annu. Rev. Mater. Sci..

[17] Baumeister J., Banhart J. (1997). Weber M. Aluminium foams for transport industry. Mater. Des..

[18] Barchi L., Bardi U., Caporali S., Fantini M., Scrivani A., Scrivani A. (2010). Electroplated bright aluminium coatings for anticorrosion and decorative purposes. Prog. Org. Coat..

[19] Ma J., He Y.D., Wang J., Sun B.D. (2008). High temperature corrosion behavior of microcrystalline aluminide coatings by electro-pulse deposition. Trans. Nonferrous Met. Soc. China.

[20] Lu J., Do I., Drzal L.T., Worden R.M., Lee I. (2008). Nanometal-decorated exfoliated graphite nanoplatelet based glucose biosensors with high sensitivity and fast response. ACS Nano.

[21] Boonyongmaneerat Y., Schuh C.A., Dunand D.C. (2008). Mechanical properties of reticulated aluminum foams with electrodeposited Ni–W coatings. Scr. Mater..

[22] Li Z.D., Huang Y.J., Wang X.F., Wang X.F., Wang D., Han F.S. (2016). Enhancement of open cell aluminum foams through thermal evaporating Zn film. Mater. Lett..

[23] Liu J., Zhu X.Y., Jothi S., Diao W., Yu S. (2011). Increased Corrosion Resistance of Closed-Cell Aluminum Foams by Electroless Ni-P Coatings. Mater. Trans..

[24] Leszczyńska A., Winiarski J., Szczygiel B., Szczygiel I. (2016). Electrodeposition and characterization of Ni–Mo–ZrO_2_ composite coatings. Appl. Surf. Sci..

[25] Alizadeh M., Cheshmpish A. (2019). Electrodeposition of Ni-Mo/Al_2_O_3_ nano-composite coatings at various deposition current densities. Appl. Surf. Sci..

[26] Beltowska-Lehman E., Indyka P. (2012). Kinetics of Ni–Mo electrodeposition from Ni-rich citrate baths. Thin Solid Film.

[27] Chang C.S., Hou K.H., Ger M.D., Chung C.K., Lin J.F. (2016). Effects of annealing temperature on microstructure, surface roughness, mechanical and tribological properties of Ni–P and Ni–P/SiC films. Surf. Coat. Technol..

[28] Zhou Y., Xie F.Q., Wu X.Q., Zhao W.D., Chen X. (2017). A novel plating apparatus for electrodeposition of Ni-SiC composite coatings using circulating-solution co-deposition technique. J. Alloy Compd..

[29] Kumar K.A., Kalaignan G.P., Muralidharan V.S. (2012). Pulse and Pulse Reverse Current Electrodeposition and Characterization of Ni–W–TiN Composites. Sci. Adv. Mater..

[30] Chen F.C., Liu X.J. (2014). Study on Electrodeposition of Ni-Mo Alloy. J. Hunan Univ. Nat. Sci..

[31] Antenucci A., Guarino S., Tagliaferri V., Ucciardello N. (2014). Improvement of the mechanical and thermal characteristics of open cell aluminum foams by the electrodeposition of Cu. Mater. Des..

[32] Li B.S., Li X., Huan Y.X., Xia W.Z., Zhang W.W. (2018). Influence of alumina nanoparticles on microstructure and properties of Ni-B composite coating. J. Alloy Compd..

[33] Smith B.H., Szyniszewski S., Hajjar J.F., Schafer B.W., Arwade S.R. (2012). Steel Foam for Structures: A Review of Applications, Manufacturing and Material Properties. J. Constr. Steel Res..

[34] Jang W.Y., Kyriakides S., Kraynik A.M. (2010). On the compressive strength of open-cell metal foams with Kelvin and random cell structures. Int. J. Solids Struct..

[35] Lan F.C., Zeng F.B., Zhou Y.J., Chen J.Q. (2014). Progress on Research of Mechanical Properties of Closed-cell Aluminum Foams and Its Applications in Automobile Crashworthiness. Chin. J. Mech. Eng..

[36] Gibson L.J., Ashby M.F. (1999). Cellular Solids Structures and Properties-Second Edition.

[37] Li N., Gao C.H. (2011). Microstructure and electrochemical properties of the electrodeposited Ni-Mo/ZrO_2_ Alloy coating. Mater. Sci. Technol. Lond..

[38] Xu Y., Ma S., Fan M.Y., Chen Y., Song X., Hao J. (2019). Design and properties investigation of Ni-Mo composite coating reinforced with duplex nanoparticles. J. Surf. Coat. Technol..

